# Contact exposure to neonicotinoid insecticides temporarily suppresses the locomotor activity of *Pardosa lugubris* agrobiont wolf spiders

**DOI:** 10.1038/s41598-022-18842-0

**Published:** 2022-08-30

**Authors:** Milan Řezáč, Gabriela Přibáňová, Nela Gloríková, Petr Heneberg

**Affiliations:** 1 Crop Research Institute, Prague, Czech Republic; 2 Third Faculty of Medicine, Charles University, Ruská 87, 100 00 Prague, Czech Republic

**Keywords:** Zoology, Toxicology

## Abstract

Exposure to numerous chemicals disrupts the spiders' locomotion. Spiders, particularly epigeic spiders, are dependent on their locomotory activities to search for prey, hide from their enemies, and perform sexual reproduction and subsequent parental care. Among the best-known compounds that inhibit the locomotion of arthropods are neonicotinoids. Despite spiders are less affected by the neonicotinoids than insects due to the sequence differences in their acetylcholine receptors, they are not resistant to these compounds. We hypothesized that acute exposure to a broad spectrum of neonicotinoids suppresses the traveled distance, mean velocity, and maximum velocity in epigeic spiders. As a model species, we used adults of *Pardosa lugubris*. We tested commercial formulations of thiamethoxam, acetamiprid, and thiacloprid. We tested each of the neonicotinoids in the maximum and minimum concentrations recommended for foliar applications. We applied them under controlled conditions dorsally by spraying them directly on the spiders or exposing the spiders to the tarsal contact with neonicotinoid residues. Control groups consisted of 31 individuals; treated groups consisted of 10–21 individuals. We found that a broad spectrum of neonicotinoids temporarily suppresses the traveled distance in epigeic spiders. At 1 h after application, all the three tested neonicotinoid insecticides induced declines in the traveled distance, but this effect mostly disappeared when tested at 24 h after the application. The decrease in the traveled distance was associated with substantial temporary decreases in the mean and maximum velocities. Despite differences among modalities, all three insecticides caused multiple adverse effects on the locomotory parameters in any tested concentrations. It remains to test what would be the lowest safe concentration for the chronic exposure to neonicotinoids in epigeic spiders.

## Introduction

Spider populations reach high densities in European crop fields^1^. In central Europe, crop field margins are dominated by Lycosidae^2,3^, whereas Linyphiidae dominates these habitats in Western Europe and also dominates centers of larger field blocks^1^. In crop fields, the lycosids prey on Aphidoidea, Diptera, and Collembola^4^. Removal of spiders leads to an increased abundance of pest aphids^5–7^. In agrocenoses, the spiders are subject to both acute and chronic exposures to pesticides. Some active compounds degrade within days^8^, but other compounds, including some neonicotinoids, may remain for many months or even years^9,10^. The observed effects may therefore vary from lethal and sublethal effects to hormetic and even transgenerational hormetic effects^11,12^. The effects on biocontrol agents, such as spiders, could be direct but also indirect through the exposure of the prey. The indirect effects may also include habitat changes^13^, sublethal and hormetic effects in key pest prey^11,12^, resistance development^14^, and changes in food webs and subsequent secondary pest outbreaks that require the application of additional integrated management measures^15^.

Spiders, particularly epigeic spiders, depend on their locomotory activities to search for prey, hide from their enemies, and perform sexual reproduction and subsequent parental care. Spider locomotion is disrupted by exposure to numerous chemical compounds. The reasons why locomotion is disrupted remain insufficiently understood. In addition, the spectrum of species affected and compounds causing these effects remain underresearched. Interestingly, neurotoxic compounds, like organophosphorus, carbamate, cyclodiene, and pyrethroid formulations, cause accelerated water loss^16^. They likely target humidity signaling in the cuticle. Therefore, spiders stop searching for a more favorable environment due to locomotion disruption and increased water excretion^16^. Pyrethroids cause temporary relaxation of flexor muscles^17,18^ and induce complete paralysis or reduced movement velocity^16–19^. The exposition to pyrethroids and the organophosphates also causes uncoordinated walking patterns^20^. Under certain circumstances, these compounds may also induce increased movement, indicating avoidance by repulsion and subsequent dispersal^21–23^. The data on the effects of neonicotinoids on spider locomotion are scarce. Dorsal contact treatment of *Hogna antelucana* (Lycosidae) by three neonicotinoid compounds decreased the total distance traveled and velocity^24^. The neonicotinoids target nicotinic acetylcholine receptors. Earlier studies suggested that, unlike in vertebrates, acetylcholine is not the principal neurotransmitter at the neuromuscular junction of arthropods, and nicotinic acetylcholine receptors are exclusively located in the central nervous system^25^. However, more recently, a motor neuron function of the nicotinic acetylcholine receptors *nAChRα1* and *nAChRα3* was suggested, and inactivation of *nAChRα1* and *nAChRα3* in the neurons caused significant movement defects in the *Drosophila* model^26^. This is in line with earlier observations that cholinergic input directly stimulates motor neurons^27^. Therefore, the effects of neonicotinoids on the locomotor activity of arthropods need to be analyzed.

The neonicotinoids are well-known for their adverse effects on the diversity of invertebrates, particularly bees^28–32^. Spiders have a different structure of acetylcholine receptors, which makes them less affected, but numerous sublethal effects and increased lethality were reported^33–36^. The effects of neonicotinoids were initially described in honey bees *Apis mellifera*^37,38^ and later confirmed in other bees, like *Tetragonisca angustula*^39^ and bumblebees^40,41^. Other invertebrates are affected as well; previous reports include *Drosophila* flies^42^, *Platynus* carabid beetles^43^, and even the *Caenorhabditis* nematodes^44,45^. As stated above, only one study is available on the effects of neonicotinoids on spider locomotion. This study reported adverse effects, with favorable results reported for thiamethoxam relative other two tested neonicotinoids but focused on a single spider species, did not test tarsal application, measured only immediate effects (here, we also tested effects after 24 h following the application), and did not analyze the maximum velocity^24^.

In the present study, we hypothesized that acute exposure to a broad spectrum of neonicotinoids suppresses the traveled distance, mean velocity, and maximum velocity in epigeic spiders. We tested the formulations of three neonicotinoid insecticides broadly used in foliar applications and applied them by two modes of contact. These mimic the situation in agrocenoses—the epigeic spiders may be directly sprayed during the crop treatment, or, if they manage to hide during the application of neonicotinoids, they may be in contact with the residues present on the soil and plant surfaces. As a model species, we used adults of *Pardosa lugubris* wolf spiders, an abundant spider of agrocenoses with the Palearctic distribution^46–48^.

## Materials and methods

### Model organism

As a model organism, we used adult *P. lugubris* wolf spiders. We collected them at an arable field near Vodokrty, Czech Republic (45.59°N, 13.39°E; 415 m a.s.l.). We placed the spiders individually into vials with carbon plaster on the bottoms and enclosed them with foam plugs. We stored the spiders before their use in laboratory thermostats, maintaining temperatures between 5 °C and 15 °C, 80% humidity, and a natural light/dark cycle.

### Tested neonicotinoids

We tested three neonicotinoids in formulations and concentrations commonly sprayed on crops to eliminate pest insects. These included commercial formulations of thiamethoxam, acetamiprid, and thiacloprid. We used a 25% formulation of thiamethoxam, known as Actara 25 WG (Syngenta Crop Protection, Basel, Switzerland), with a suggested application rate of 70–80 ml ha^−1^. We used a 20% formulation of acetamiprid, formulated as Mospilan 20 SP (Nippon Soda Co., Tokyo, Japan), which has a suggested application rate of 60–250 ml ha^−1^. Further, we used a 22.97% formulation of thiacloprid, formulated as Biscaya 240 OD (Bayer CropScience, Monheim, Germany), with a suggested application rate of 200–300 ml ha^−1^. Thiacloprid is actively used in the United States (mainly on cotton and fruits) and other countries but has been recently banned in the European Union. As a vehicle and mock control, we used distilled water.

We tested 268 spiders. The treatment groups consisted of 31 individuals (each mock-treated group), 18–21 individuals (groups tested with high concentrations of the compounds), and 10–12 individuals (groups tested with low concentrations of the compounds). The slight differences in the numbers of tested individuals were caused by the partial lethality of the study compounds. All the spiders were assigned to only a single treatment group and were not exposed repeatedly.

We applied all the compounds in a volume equal to 400 L ha^-1^ using the Potter Precision Laboratory Spray Tower (Burkard Scientific, Uxbridge, UK). The applied doses were 175 mg L^−1^ and 200 mg L^−1^ for thiamethoxam, 150 mg L^−1^ and 625 mg L^−1^ for acetamiprid Mospilan, 478.54 mg L^−1^, and 717.81 mg L^−1^ for thiacloprid. We dissolved the commercial formulations in distilled water to reach the desired concentrations. The applied concentrations were derived from concentrations recommended for using these compounds in foliar applications in agriculture.

We used two modes of application of the study compounds, topical and tarsal. For the topical application, we sprayed the neonicotinoids or distilled water directly onto the dorsal side of the spiders that were already present individually in wells of 12-well plates. For the tarsal application, we sprayed the neonicotinoids or distilled water in empty 12-well plates, and then the spiders were inserted by an exhaustor into the individual wells. The spiders were allowed to be present in the 12-well plates for one hour (this applies both for the dorsal and tarsal applications). We covered the plates with a mesh instead of a standard plastic lid to allow evaporation of the vehicle. At the end of the one-hour window, we recorded the acute mortality and subjected the surviving spiders to the follow-up locomotion experiments.

### Locomotor parameters

One hour after treatment with neonicotinoids or distilled water, we moved the spiders to clean 12-well plates and placed them individually into the available wells. The wells were plugged with a translucent plug to avoid the escape of the spiders. We videotaped the spiders in their 3.8 cm^2^ arenas (wells of 12-well plates) for 10 min with the Panasonic WV-CP480 SDIII-Super Dynamic camera. After the videotaping session, we placed the spiders into 1.5 mL tubes and stored them at 10–15 °C and 80% humidity in a laboratory thermostat. After 24 h, we removed the spiders from the thermostat, recorded the mortality, and placed the living spiders in the arenas again. We then performed a second 10-min videotaping session. We used the tracking software EthoVision to measure the behavior of spiders during the video-recorded trials^49^. We compared the total distance moved [cm], mean velocity [mm s^−1^], and maximum velocity [mm s^−1^].

### Data analysis

We used the generalized linear models (GLM) test for differences in the traveled distance in insecticide-treated and mock-treated spiders (quasi-Poisson settings). We employed one-sided *t*-tests to analyze the differences in responses to individual compounds and concentrations. Data are shown as the mean ± SE unless stated otherwise. We performed all statistical analyses in the R environment.

## Results

### Distance traveled

The distance traveled was significantly affected by applying the maximum concentrations of the neonicotinoids at *t* = 1 h after their application. The GLM with a quasi-Poisson setting revealed significant effects of the applied compounds and water (deviance 515.22, Df 4, *p* < 0.001). There were also differences between the spiders' responses to the topical and tarsal application (deviance 122.33, Df 1, *p* < 0.001). However, the interaction between the tested compounds and the mode of application was not significant (deviance 85.17, Df 4, *p* = 0.09). The ANODEV table for the respective GLM test is provided in Table 1a.

**Table 1:** ANODEV tables for GLM with quasi-Poisson setting testing the null hypothesis that the tarsal and topical application of the neonicotinoid insecticides on the total distance traveled by the treated spiders did not differ with treatment compounds and between treatment modes after 1 h (a) and 24 h (b).

	Df	Deviance	Resid. Df	Resid. Dev	*P* (> Chi)
**a—1 h**					
NULL			263	3390.1	
Compound	4	515.22	159	2874.9	**6.1 e−10**
Mode	1	122.33	258	2752.6	**0.0007**
Compound:Mode	4	85.17	254	2667.4	0.09
**b—24 h**					
NULL			271	2601.5	
Compound	4	187.23	267	2414.2	**0.0004**
Mode	1	5.84	266	2408.4	0.42
Compound:Mode	4	18.52	262	2389.9	0.73

Comparisons of residuals versus fitted, SD of residuals vs. theoretical quantiles and predicted values, and Cook`s distance are provided in Fig. S1.

Significant values are in bold

Later, 24 h after applying the maximum concentrations of the neonicotinoids, the effects on the distance traveled were less prominent. The GLM with a quasi-Poisson setting revealed that there continued to be significant effects of the applied compounds and water (deviance 187.23, Df 4, *p* < 0.001), but the differences between the tarsal and dorsal modes of application disappeared (deviance 5.84, Df 1, *p* = 0.42). There were also no effects of the interaction between the tested compounds and the mode of application (deviance 18.52, Df 4, *p* = 0.73). The ANODEV table for the respective GLM test is provided in Table 1b.

**Figure 1:**
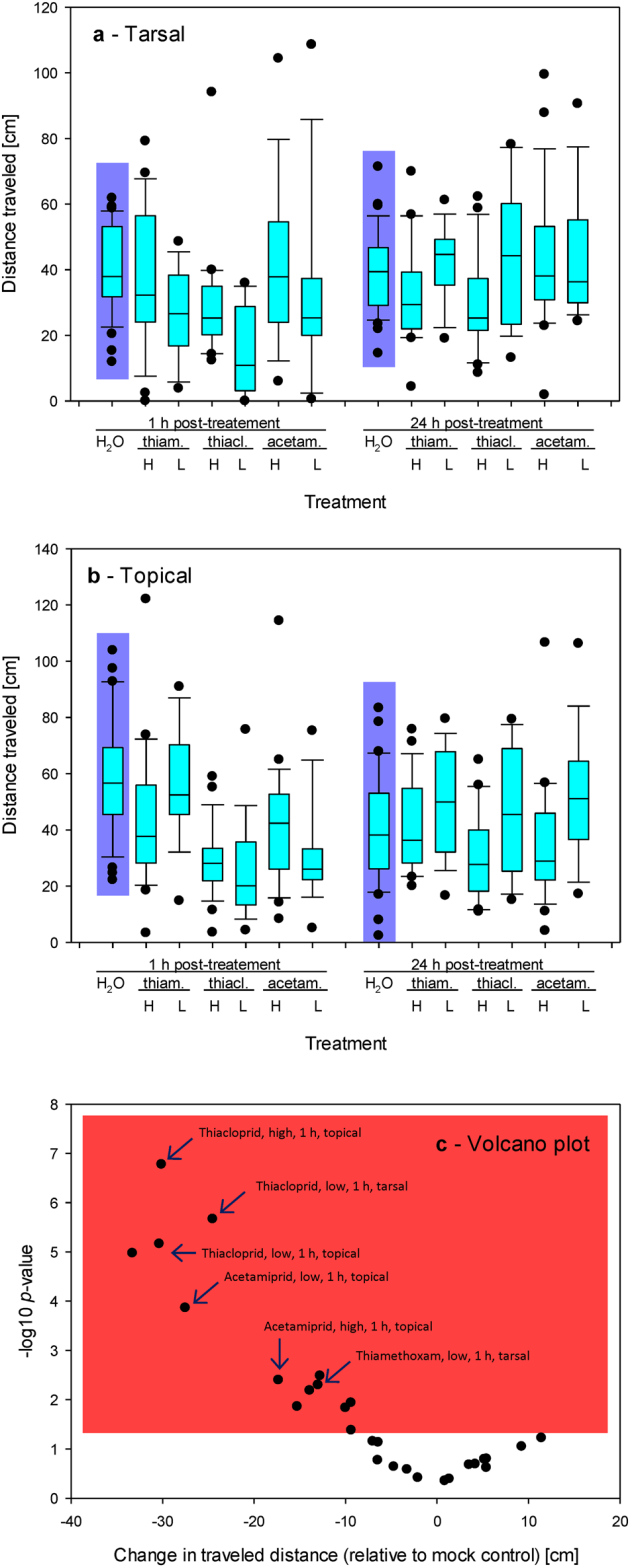
Changes in traveled distance in response to the treatment with neonicotinoids. (**a**) Tarsal application. (**b**) Topical application. (**a**–**b**) Abbreviations: thiam. = thiamethoxam, thiacl. = thiacloprid, acetam. = acetamiprid, H = the highest recommended concentration, L = the lowest recommended concentration, 1 = data measured at 1 h after the treatment; 24 = data measured at 24 h after the treatment. (**c**) Volcano plot showing − log10 of the probability values resulting from the comparison of treated groups with the mock control by one-sided *t*-tests and the corresponding differences between the treated groups and the mock control. The plot contains the data collected at 1 h after the treatment and 24 h after the treatment; the data were analyzed separately, and each has its mock control group.

In some cases, the distance traveled was differentially affected by the two concentrations of the neonicotinoid insecticides used. When treated tarsally with thiamethoxam, the distance traveled remained similar 1 h and 24 h after the treatment with high thiamethoxam concentration but increased for the low thiamethoxam concentrations. Therefore, 24 h after the treatment, the two concentrations differed significantly in their effects (32.6 ± 4.0 cm vs. 42.6 ± 3.4 cm; *t*-test *t* -1.750, Df 26, *p* < 0.05). Similarly, the distance traveled after the treatment with thiacloprid remained similar at 1 h and 24 h after the exposure to high thiacloprid concentration. However, the treatment with low thiacloprid concentrations resulted initially in a much shorter traveled distance (30.1 ± 4.0 cm vs. 15.6 ± 3.9 cm; *t*-test *t* − 2.391, Df 29, *p* = 0.01), but then the spiders quickly recovered and traveled a longer distance compared to their counterparts treated with high thiacloprid concentration at t = 24 h (29.7 ± 3.4 cm vs. 44.5 ± 6.0 cm; *t*-test *t* − 2.231, Df 29, *p* < 0.05). We observed a similar but much milder trend in spiders treated tarsally with acetamiprid, but the effect was not significant (*t*-test *t* 0.605, Df 21, *p* > 0.05 at high thiacloprid, and *t* 0.120, Df 29, *p* > 0.05 at low thiacloprid, respectively) (Fig. 1a).

When we used the topical application, we observed similar trends. The spiders treated topically with a high concentration of thiamethoxam traveled a similar distance at 1 h and 24 h after the treatment. But there was a trend toward a longer distance traveled in spiders treated topically with a low concentration of thiamethoxam; the difference was, however, not significant (*t*-test *t* 0.244, Df 35, *p* > 0.05 at *t* = 1 h, and *t* 0.847, Df 22, *p* > 0.05 at *t* = 24 h, respectively). The spiders treated topically with a high concentration of thiacloprid traveled a similar distance at *t* = 1 h and *t* = 24 h. However, the treatment with low thiacloprid concentration resulted initially in a decline in traveled distance similar to spiders treated with the high concentration of the same compound, but then the spiders quickly recovered and traveled longer distance compared to their counterparts treated with high thiacloprid concentration at t = 24 h (31.0 ± 3.5 cm vs. 45.8 ± 6.9 cm; *t*-test *t* 2.061, Df 28, *p* < 0.05). Similarly, the spiders treated topically with a high concentration of acetamiprid traveled a similar distance at *t* = 1 h and *t* = 24 h. The treatment with low acetamiprid concentration resulted initially in decline similar to a decline in traveled distance similar to spiders treated with the high concentration of the same compound. However, the spiders recovered and traveled longer distances compared to their counterparts treated with high acetamiprid concentration at *t* = 24 h (35.7 ± 5.3 cm vs. 51.8 ± 6.7 cm; *t*-test *t* 1.837, Df 28, *p* < 0.05) (Fig. 1b).

The tarsal application of thiamethoxam and thiacloprid generally led to a decrease in traveled distance or no change in some application modes. In contrast, the tarsal application of acetamiprid did not induce any significant changes in the traveled distance (Fig. 1a). When we administered the same compounds topically, all three tested compounds induced declines in the traveled distance when tested at 1 h after the application. The effects mostly disappeared when tested 24 h after the application (Fig. 1b). Raw data are shown in Table S1.

### Mean velocity

**Figure 2:**
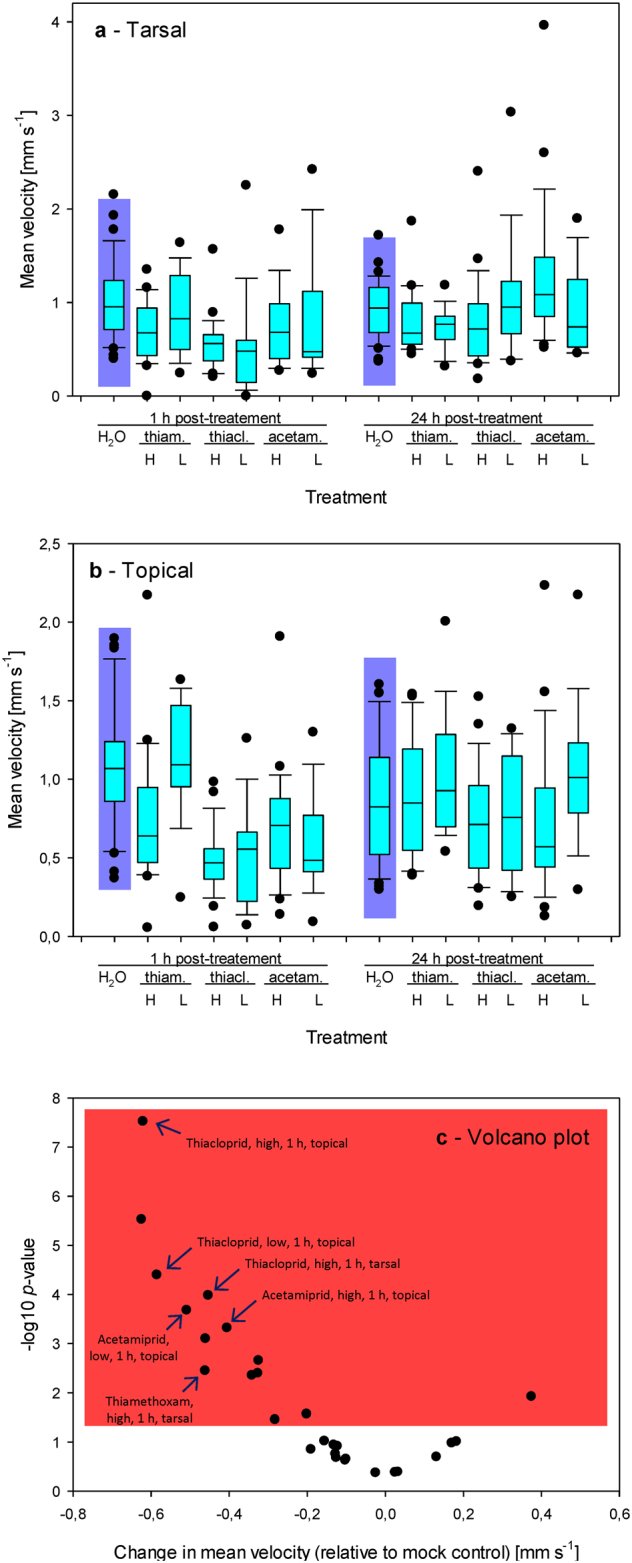
Changes in mean velocity in response to the treatment with neonicotinoids. (**a**) Tarsal application. (**b**) Topical application. (**a**, **b**) Abbreviations: thiam. = thiamethoxam, thiacl. = thiacloprid, acetam. = acetamiprid, H = the highest recommended concentration, L = the lowest recommended concentration, 1 = data measured at 1 h after the treatment; 24 = data measured at 24 h after the treatment. (**c**) Volcano plot showing -log10 of the probability values resulting from the comparison of treated groups with the mock control by one-sided *t*-tests and the corresponding differences between the treated groups and the mock control. The plot contains the data collected at 1 h after the treatment and 24 h after the treatment; the data were analyzed separately, and each has its mock control group.

Mean velocity was insensitive to the concentrations used for tarsal applications (*t*-test *p* > 0.05 for each comparison; Fig. 2a). We also observed similar values for both concentrations used except for thiamethoxam when using the topical application. Topically applied thiamethoxam induced more severe effects on the mean velocity at 1 h after the treatment when applied at a higher concentration (0.75 ± 0.10 mm s^-1^ vs. 1.13 ± 0.10 mm s^-1^; *t*-test *t* -1.381, Df 27, *p* = 0.01). This difference faded out at *t* = 24 h (0.89 ± 0.09 mm s^-1^ vs. 1.04 ± 0.12 mm s^-1^; *t*-test *t* 0.648, Df 26, *p* = 0.17) (Fig. 2b).

The tarsal application of all the three neonicotinoids led to decreases in mean velocity at 1 h after the treatment, except for a low concentration of acetamiprid. The spiders quickly recovered and ran at equal or insignificantly lower mean velocity 24 h after the treatment. In addition, the spiders treated with a high concentration of acetamiprid displayed significantly higher velocity at 24 h after the treatment compared to the mock control (Fig. 2a,c).

Similarly, the topical application of all the three neonicotinoids led to decreases in mean velocity at 1 h after the treatment, except for a low concentration of thiamethoxam. In all cases, the spiders quickly recovered, and their mean velocity at 24 h after the treatment was similar to the mock control (Fig. 2b,c). Note that when we treated the spiders topically with the recommended concentration of imidacloprid, we did not observe the recovery in terms of a change in mean velocity between 1 and 24 h after the treatment. Raw data are shown in Table S1.

### Maximum velocity

**Figure 3:**
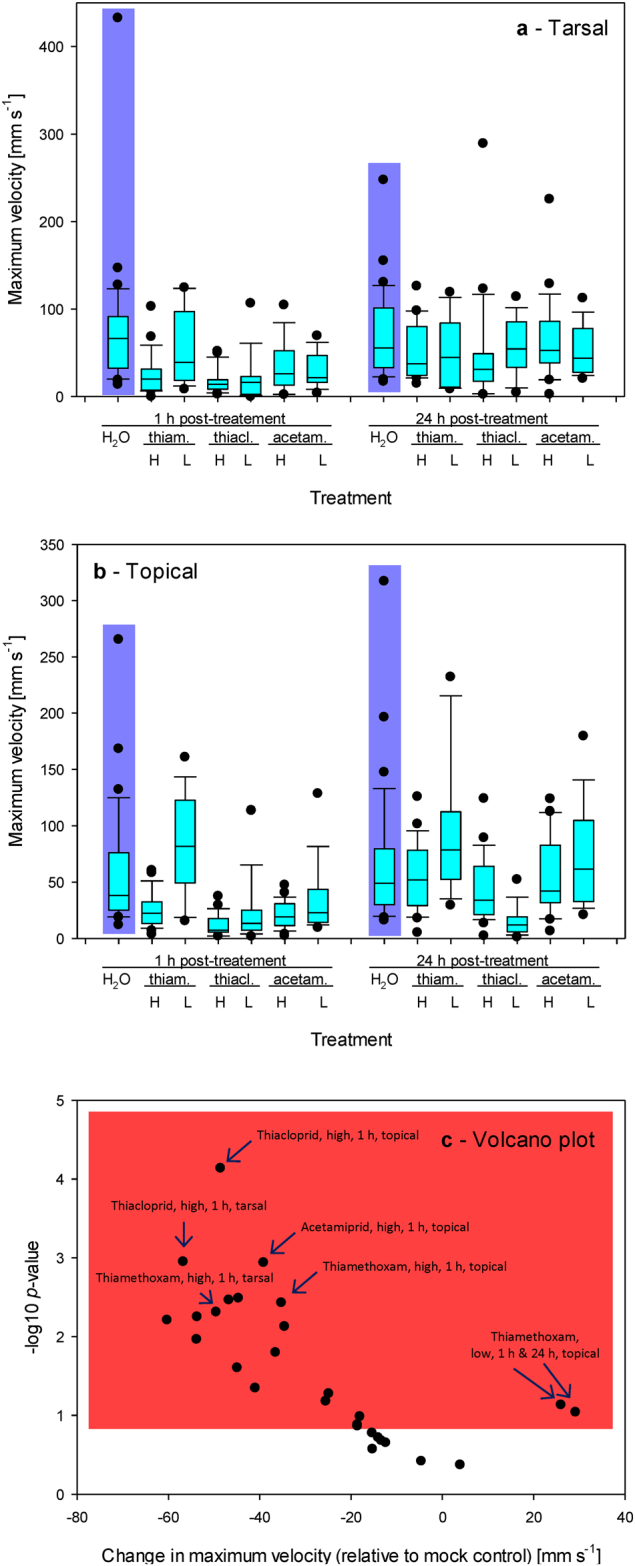
Changes in maximum velocity in response to the treatment with neonicotinoids. (**a**) Tarsal application. (**b**) Topical application. (**a**, **b**) Abbreviations: thiam. = thiamethoxam, thiacl. = thiacloprid, acetam. = acetamiprid, H = the highest recommended concentration, L = the lowest recommended concentration, 1 = data measured at 1 h after the treatment; 24 = data measured at 24 h after the treatment. (**c**) Volcano plot showing − log10 of the probability values resulting from the comparison of treated groups with the mock control by one-sided *t*-tests and the corresponding differences between the treated groups and the mock control. The plot contains the data collected at 1 h after the treatment and 24 h after the treatment; the data were analyzed separately, and each has its mock control group.

The tarsal application of neonicotinoids substantially decreased maximum velocity, but the response was not concentration-dependent (*t*-test *p* > 0.05 for each comparison) except for 1 h response to thiamethoxam (26.0 ± 5.7 mm s^−1^ vs. 60.2 ± 13.3 mm s^-1^, *t*-test *t* -2.602, Df 27, *p* = 0.007) (Fig. 3a). When treated topically, the differences were more prominent.

The difference between applied concentrations of topically applied thiamethoxam was significant at 1 h after the treatment (24.9 ± 3.5 mm s^−1^ vs. 86.2 ± 13.1 mm s^−1^, *t* -5.223, Df 29, *t*-test *p* < 0.001) and remained significant at 24 h after the treatment (54.9 ± 7.0 mm s^−1^ vs. 98.1 ± 17.7 mm s^-1^, *t*-test *t* − 2.481, Df 28, *p* < 0.01). Similarly, the differences in maximum velocity of spiders treated topically with thiacloprid were significant at 1 h (11.6 ± 2.1 mm s^−1^ vs. 23.6 ± 8.5 mm s^−1^, *t*-test *t* − 1.666, Df 31, *p* = 0.05). At 24 h after the treatment, the spiders treated with low thiacloprid concentrations did not recover. However, the recovery was present in those treated with high thiacloprid concentration (15.2 ± 4.4 mm s^−1^ vs. 44.0 ± 6.5 mm s^−1^, *t*-test *t* − 4.667, Df 39, *p* = 0.004). The differences in the maximum velocity of spiders treated topically with acetamiprid were significant at 1 h after the treatment (21.0 ± 2.6 mm s^−1^ vs. 34.7 ± 9.3 mm s^−1^, *t*-test *t* − 1.654, Df 30, *p* = 0.05). Still, the spiders recovered more quickly than after other treatments, and the maximum velocity at 24 h after the treatment did not differ between the doses used (55.5 ± 8.5 mm s^−1^ vs. 72.8 ± 13.3 mm s^−1^, *t*-test *t* − 1.113, Df 28, *p* = 0.14) (Fig. 3b).

The tarsal application of all the three neonicotinoids led to decreases in maximum velocity at 1 h after the treatment, except for a low concentration of acetamiprid. The spiders quickly recovered and ran at equal or insignificantly lower maximum velocity 24 h after the treatment (Fig. 3a,c). Note that when we treated the spiders tarsally with the recommended concentration of imidacloprid, we observed much milder recovery in terms of a change in maximum velocity between 1 and 24 h after the treatment (15.2 ± 3.1 mm s^−1^ vs. 35.4 ± 6.6 mm s^−1^, *t*-test against mock control *t* -2.621, Df 40, *p* < 0.01 at *t* = 1 h, and *t* -2.532, Df 45, *p* < 0.01 at t = 24 h, respectively).

Similarly, the topical application of all the three neonicotinoids led to decreases in maximum velocity at 1 h after the treatment except for low concentrations of thiamethoxam and acetamiprid. The spiders quickly recovered, and their maximum velocity at 24 h after the treatment was similar to in the mock control except for thiacloprid-treated spiders, which failed to recover (Fig. 3b,c). Note that when we treated the spiders topically with the recommended concentration of imidacloprid, we also did not observe the recovery in terms of a change in maximum velocity between 1 and 24 h after the treatment (15.5 ± 2.0 mm s^−1^ vs. 22.2 ± 3.4 mm s^−1^, *t*-test against mock control *t* − 2.868, Df 41, *p* < 0.01 at *t* = 1 h, and *t* − 2.840, Df 43, *p* < 0.01 at *t* = 24 h, respectively). Raw data are shown in Table S1.

## Discussion

We found that a broad spectrum of neonicotinoids temporarily suppresses the distance traveled by epigeic spiders. At 1 h after application, all the three tested neonicotinoid insecticides induced declines in the traveled distance, but this effect mostly disappeared when tested at 24 h after the application. The decrease in the traveled distance was associated with substantial temporary decreases in the mean and maximum velocities. There were differences among the tested compounds, and there were settings under which one of the three tested compounds did not cause significant effects. Despite that, all three insecticides caused multiple adverse effects on the locomotory parameters in any tested concentrations. Therefore, the neonicotinoids affect epigeic spiders when they are directly sprayed during the crop treatment or when they manage to hide during the application of the neonicotinoids and become in contact with the residues present on the soil and plant surfaces only.

The observed effects were similar to those reported by Řezáč et al. in neonicotinoid-treated *H. antelucana*^24^. In this spider, they also observed decreases in the mean velocity of affected individuals and traveled distance. They treated *H. antelucana* only topically and observed the behavior at 1 h after the treatment. They found that thiamethoxam had the weakest effect on the mean velocity. Still, the distance traveled was affected by thiamethoxam to a similar extent as thiacloprid. However, acetamiprid had the most potent effect^24^. In the present study, we see thiamethoxam having weaker effects under specific settings. Still, the mean and maximum velocities were significantly shortened when we used the highest recommended concentration of this compound (Figs. 2, 3). When we applied thiamethoxam at the lowest recommended concentration, there were no significant effects on the distance traveled or the velocities when applied topically. However, some effects were still detectable when applied tarsally (Figs. 1, 2, 3).

Surprisingly, all three tested neonicotinoids caused adverse effects on locomotor parameters. These effects can be observed when using both the lowest and highest recommended concentrations and in both modes of administration. In many cases, some of the effects were not detectable or faded within several hours after the treatment. However, there was no safe application mode and no safe dose. Most of the effects faded out; however, we found a more prolonged response associated with thiacloprid and imidacloprid. However, this report is relevant to acute, short-term exposure to neonicotinoids. In nature, the half-life of neonicotinoids in the soil may exceed 1000 days, and they may persist in the plants for over a year^50^. Therefore, in neonicotinoid-treated fields and neonicotinoid-treated orchards, the spiders are chronically exposed to neonicotinoids. Therefore the observed effects could persist not only for several hours but several days, which would be detrimental to their fitness. In bees, the topical treatment with nitro-containing neonicotinoids (imidacloprid or thiamethoxam) has more adverse effects compared to the cyano-group-containing ones (acetamiprid and thiacloprid)^51,52^. However, we did not observe any such difference between the representants of these two groups of neonicotinoids. Contact exposure of bees to low concentrations of imidacloprid and acetamiprid increased locomotor activity. Only high concentrations were inhibitory^53^. In the present study, we did not observe any consistent increases in locomotor activity at low doses of the tested neonicotinoids (Figs. 1, 2, 3). Other studies on bees reported the absence of adverse effects of acetamiprid and thiamethoxam on the locomotor activity of bees^54,55^. However, in the experimental settings of the present study, these two neonicotinoids had adverse effects on various locomotor activity parameters.

Further experiments should address the management implications of altered spider locomotion. Locomotion performances are important features allowing to disperse, migrate, overwinter, colonize new regions (and re-colonize the crop fields), and hunt prey. These features are critical when exposed to poor or disturbed environments, including the annual disturbance cycles in crop fields, food shortages, and high intraspecific density^56^. Many species, including spiders, experience repeated extinctions or migrations of a significant part of the populations in crop fields and other agroecosystems^57–60^. Therefore, the locomotion-driven recolonization from surrounding refuges is essential for the presence of spiders in these habitats^61–64^. The colonization of large crop field blocks is challenging. The crop fields serve mainly as a partial habitat for the study species, *P. lugubris*^65^, while it prefers forest margins for overwintering in leaf litter^66^. Interestingly, this species is nearly absent from crop fields in Western Europe, but it re-colonizes them once left as set-aside fields^67^. The recolonization of set-aside fields also indirectly implies the limitation by post-disturbance locomotion or the effects of temporary withdrawal of yet unidentified agrochemicals. Here, we found that the tested neonicotinoids alter the distance traveled and velocity of the study species. As the presence of *P. lugubris* in crop fields depends on the annual recolonization of these habitats, we speculate that the withdrawal of neonicotinoid insecticides may improve the recolonization abilities of *P. lugubris*. A large part of the observed effects was temporary but was induced not only by the direct (topical) application of the neonicotinoids but also by tarsal exposure to their residues. Therefore, we speculate that prolonged tarsal exposure may lead to the chronic manifestation of adverse effects on *P. lugubris* locomotion.

In conclusion, acute exposure to all the tested neonicotinoids, their topical application or contact with residues, and two concentrations representing their maximum and minimum concentrations for foliar applications induced adverse effects on locomotor activity of *P. lugubris*. The intensity of effects varied, and most of them faded after several hours following the exposure. However, as the exposure of spiders to neonicotinoid in agrocenoses is rather chronic, the effects might be more persistent than what they appear to be based on the acute tests. Therefore, their potential to shape the spider communities in agrocenoses is enormous. It remains to test what would be the lowest safe concentration for the chronic exposure to neonicotinoids in epigeic spiders.

## Supplementary Information

Supplementary Information.
